# Quasi-Distributed Temperature and Strain Sensors Based on Series-Integrated Fiber Bragg Gratings

**DOI:** 10.3390/nano12091540

**Published:** 2022-05-02

**Authors:** Huajian Zhong, Xueya Liu, Cailing Fu, Baijie Xu, Jun He, Pengfei Li, Yanjie Meng, Chao Du, Lin Chen, Jian Tang, Yiping Wang

**Affiliations:** 1 Key Laboratory of Optoelectronic Devices and Systems of Ministry of Education and Guangdong Province, College of Physics and Optoelectronic Engineering, Shenzhen University, Shenzhen 518060, China; zhj5512178@163.com (H.Z.); liuxueya2018@email.szu.edu.cn (X.L.); xubaijie2019@email.szu.edu.cn (B.X.); hejun07@szu.edu.cn (J.H.); lipengfei2022@163.com (P.L.); mengyanjie2020@email.szu.edu.cn (Y.M.); 2060453065@email.szu.edu.cn (C.D.); 2070456043@email.szu.edu.cn (L.C.); tangjian2@email.szu.edu.cn (J.T.); ypwang@szu.edu.cn (Y.W.); 2 Shenzhen Key Laboratory of Photonic Devices and Sensing Systems for Internet of Things, Guangdong and Hong Kong Joint Research Centre for Optical Fibre Sensors, Shenzhen University, Shenzhen 518060, China

**Keywords:** fiber Bragg grating, femtosecond laser, optical fiber sensor, quasi-distributed sensors

## Abstract

Two types of series-integrated fiber Bragg gratings (SI-FBGs), i.e., strong and weak SI-FBGs, were inscribed in a standard single-mode fiber (SMF) using the femtosecond laser point-by-point technology. In the SI-FBGs inscribing system, the grating pitch of each FBG and the distance between the two adjacent FBGs in the SI-FBGs can be flexibly controlled by adjusting the inscription parameters. The strong SI-FBGs with different grating pitches and the weak SI-FBGs with an identical grating pitch were employed to successfully measure the temperature distribution in a tube furnace and the strain distribution on a cantilever beam, respectively. A high spatial resolution of less than 1 mm was achieved during the distributed temperature sensing experiment. Moreover, the spatial resolution could be improved by decreasing the distance between the two adjacent FBGs, i.e., decreasing the FBG length and the space between the two adjacent FBGs. Hence, the inscribed high-quality SI-FBGs have great potential to be developed as various quasi-distributed sensors with a high spatial resolution.

## 1. Introduction

Fiber Bragg gratings (FBGs) modulated by a series of submicron-scale periodic refractive indexes in the fiber core have been widely used in various areas, such as fiber sensors [1], fiber lasers [2], and optical networks [3]. Series-integrated FBGs (SI-FBGs), i.e., an FBG array multiplexed several FBGs with identical or different Bragg wavelengths, have attracted considerable attention in the multi-parameter quasi-distributed sensing, such as ocean temperature and depth measuring [4], shape sensing using bend and torsion demodulation [5], and surface intrusion event identifying for subway tunnels [6]. Various techniques have been demonstrated to inscribe the SI-FBGs [7,8]. For example, an inscription system consisting of fiber feeding, coating removal, FBG writing, and fiber collecting has been demonstrated to inscribe ultra-weak SI-FBGs using the phase-mask method with KrF excimer laser-based on the UV photosensitive fiber [7]. Meanwhile, an online writing system, including a drawing tower and an FBG writing platform, was also demonstrated to inscribe continuous SI-FBGs via the phase mask method using periodic interference fringes of the ± 1st diffraction light to irradiate photosensitive fiber [8]. However, these two methods required the use of the photosensitive fiber and phase mask with a constant pitch, which limited their application in many areas. Compared with the phase mask method, the femtosecond laser direct writing methods, including point-to-point [9], line-by-line [10], and plane-by-plane [11] techniques, could be used to inscribe various high-quality FBGs in almost all types of fibers with or without photosensitivity. However, the femtosecond laser direct writing method has not been experimentally demonstrated to inscribe SI-FBGs. Moreover, quasi-distributed or distributed sensing systems have found great significance in measuring the temperature [12] or strain distribution profiles [13] in industrial process monitoring. However, due to the weak intensity of Raman [14], Brillouin [15], and Rayleigh scattering [16] intrinsic in fiber, the accuracy and distance of distributed sensing are limited. Thus, the quasi-distributed system by multiplexing several FBGs has attracted great attention attributing to serval orders of magnitude higher than the intrinsic scattering, resulting in a higher spatial resolution and sensing distance. Compared with optical time-domain reflectometry, the demodulation technology, i.e., optical frequency domain reflectometry (OFDR), has a higher spatial resolution for the quasi-distributed system based on the SI-FBGs sensor.

In this paper, two types of high-quality SI-FBGs, i.e., strong and weak SI-FBGs, were successfully inscribed in the standard single-mode fiber (SMF) using the femtosecond laser point-by-point technology. The distance domain reflection intensity spectrum of the strong SI-FBGs with different grating pitches and weak SI-FBGs with identical grating pitches were investigated to demonstrate the flexibility of the inscription system. Moreover, the abilities of the strong and weak SI-FBGs combined with the OFDR system to realize quasi-distributed temperature and strain sensing were studied, respectively.

## 2. Technology of Inscribing SI-FBGs

**Figure 1:**
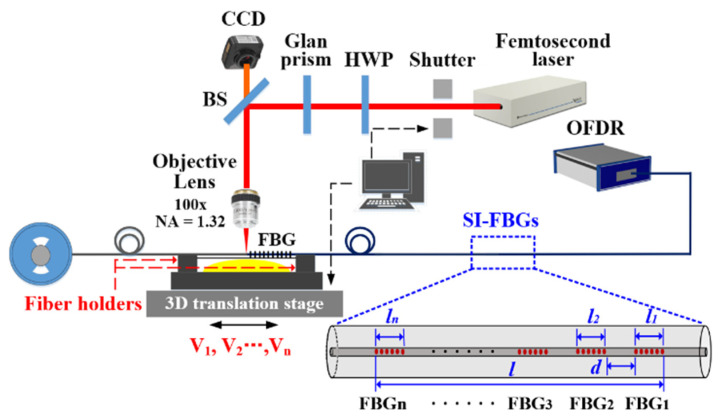
Experimental setup for inscribing online series integrated FBGs (SI-FBGs) in the standard SMF using the femtosecond laser point-by-point technology, where an OFDR was used to measure the reflection spectrum of the SI-FBGs. Inset: schematic of the inscribed SI-FBGs, i.e., FBG_1_, FBG_2_…, FBG_n_, where the lengths of the obtained SI-FBGs and single FBG were *l*, and *l_1_*, *l_2_*…, *l*_n_, respectively, and the space between the two adjacent FBGs is *d*. HWP: half-wave plate.

As shown in Figure 1, a femtosecond laser (Pharos PH1, Light Conversion) with a pulse width of 290 fs, a central wavelength of 514 nm, and a repetition rate of 200 kHz was employed to inscribe SI-FBGs in a standard SMF with a polymer coating using the point-by-point technology [17,18]. A half-wave plate (HWP) combined with a Glan prism was used to adjust the laser energy precisely. A couple of fiber holders installed on a high-precision three-dimensional translation stage were used to fix the SMF. Thus, the fiber could be moved accurately. Note that the coating of the SMF employed was not required to be pre-stripped off before the FBG was inscribed.

The femtosecond laser, the shutter, and the translation stage were simultaneously controlled to inscribe SI-FBGs in the fiber core by a LabVIEW program with a friendly operational interface. Firstly, the femtosecond laser beam was focused on the geometric center of the SMF core using an oil-immersion 100x objective lens with a numerical aperture of 1.32. Meantime, the translation stage moved the SMF along its axis with a velocity of V_1_ as soon as the shutter was opened. Thus, a series of periodic refractive-index modulations with a grating pitch of Λ_1_ were induced in the fiber core. That is, the first FBG, i.e., FBG_1_, with a length of *l_1_,* was inscribed. Secondly, the fiber was drawn by a space of *d* along its axis. Then, by repeating the inscribing step above, the second FBG, i.e., FBG_2_, with a grating pitch of Λ_2_ and a length of *l_2_* was inscribed. Finally, a series of FBGs, i.e., FBG_1_, FBG_2_…, FBG_n_, were obtained by repeating the inscribing-drawing-inscribing process above. In other words, SI-FBGs were successfully inscribed in the fiber core. Note that, during the FBG inscription process, the velocity of the translation stage along the fiber axis, i.e., V_1_, V_2_…, V_n_, could be adjusted, indicating that the grating pitch, i.e., Λ_1_, Λ_2_…, Λ_n_, of each FBG in the SI-FBGs could be identical or different.

**Figure 2:**
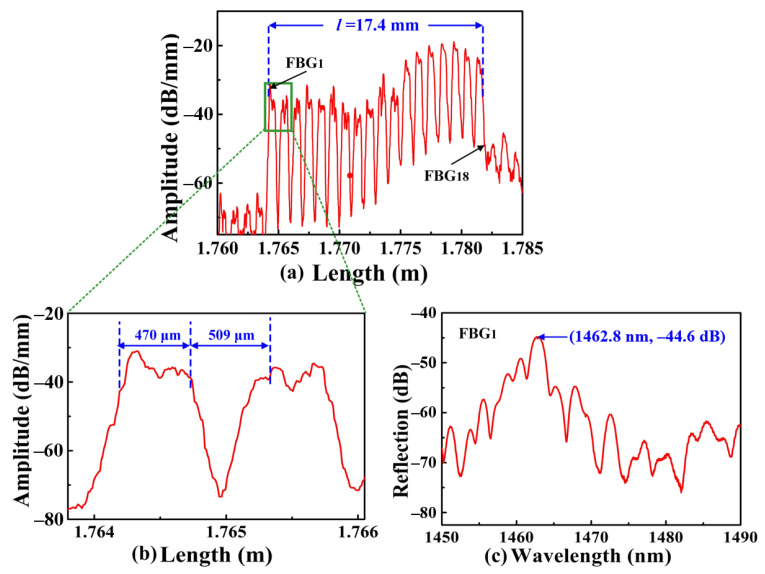
(**a**) Distance-domain reflection intensity spectrum of obtained strong SI-FBGs consisting of 18 FBGs, i.e., FBG_1_, FBG_2_…, FBG_18_, with different grating pitches of 1.010, 1.015…, 1.095 μm, respectively, measured by the OFDR; (**b**) locally enlarged view of the selected two FBGs, i.e., FBG_1_ and FBG_2_, for the strong SI-FBGs, where the grating length and the space between the two adjacent FBGs were 470, and 509 μm, respectively; (**c**) reflection spectrum of the FBG_1_ with a grating pitch of 1.010 μm.

To demonstrate the flexibility of the inscription system, two types of SI-FBGs, i.e., strong and weak SI-FBGs, were successfully inscribed in standard SMF. A strong SI-FBGs consisting of 18 FBGs, i.e., FBG_1_, FBG_2_…, FBG_18_, with different grating pitches of 1.010, 1.015…, 1.095 μm, respectively, was inscribed, where the space between the two adjacent FBGs was 509 μm, i.e., *d* = 509 μm. An OFDR was used to interrogate the position and measure the reflection spectrum of the SI-FBGs. Compared with the traditional method of using an amplified spontaneous emission light source and optical spectrum analyzer, the OFDR could measure the FBG with lower reflectivity, locate the position of the FBG, which was attributed to its high spatial resolution and sensitivity. The distance-domain reflection intensity spectrum of the strong SI-FBGs is illustrated in Figure 2. As shown in Figure 2a, each peak corresponded to one FBG, indicating that 18 FBGs were successfully inscribed in the standard SMF. Moreover, the amplitude of each FBG of the obtained SI-FBGs ranged from –38.7 to –18.9 dB/mm; thus, the obtained SI-FBGs were defined as strong SI-FBGs. The large amplitude difference of each FBG of the strong SI-FBG was attributed to the instability of femtosecond laser energy in the fabrication. The first FBG, i.e., FBG_1_, was at the position of 1.7642 m, while the last FBG, i.e., FBG_18_, was at 1.7816 m, indicating that the total length of the inscribed strong SI-FBGs was 17.4 mm, i.e., *l* = 17.4 mm. As shown in Figure 2b, the length of the single FBG was 470 μm with an equal space of approximately 509 μm between the two adjacent FBGs, which is in good agreement with the inscription parameters. Moreover, the reflection spectrum of the FBG_1_ with a grating pitch of 1.010 μm is illustrated in Figure 2c.

**Figure 3:**
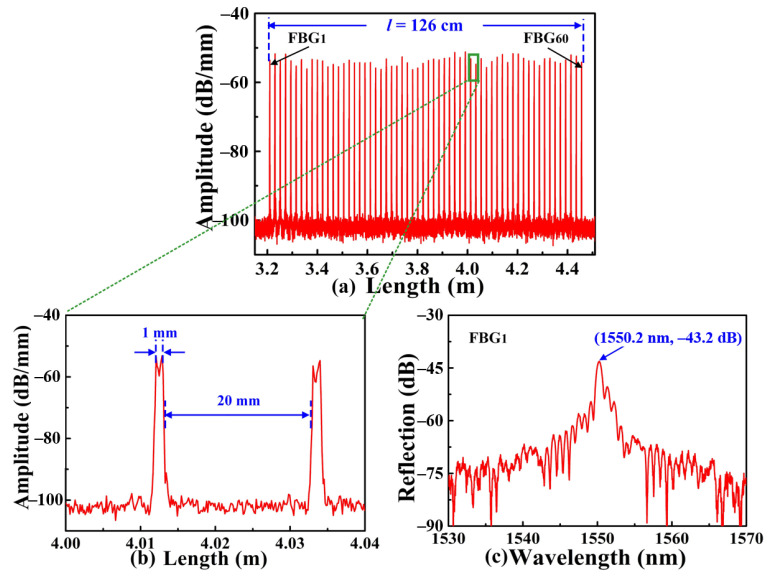
(**a**) Distance-domain reflection intensity spectrum of the obtained weak SI-FBGs consisting of 60 FBGs, i.e., FBG_1_, FBG_2_…, FBG_60_, with an identical grating pitch of 1.07 μm, respectively; (**b**) locally enlarged view of the selected two FBGs for the weak SI-FBGs, where the grating length and the space between the two adjacent FBGs were 1, and 20 mm, respectively; (**c**) reflection spectrum of FBG_1_.

As shown in Figure 3a, weak SI-FBGs consisting of 60 FBGs, i.e., FBG_1_, FBG_2_…, FBG_60_, with the identical grating pitch of 1.07 μm, were also inscribed. Compared with the afore-inscribed strong SI-FBGs, the amplitude of the weak SI-FBGs was ranged from −56.3 to −51.2 dB/mm. As shown in Figure 3b, the length of the single FBG was 1 mm with an equal space of 20 mm between the two adjacent FBGs, indicating that the length of the obtained SI-FBGs was 126 cm, i.e., *l* = 126 cm. In addition, the reflection spectrum of the FBG_1_ is illustrated in Figure 3c, where the reflection wavelength was 1550.2 nm, corresponding to the grating pitch of 1.07 μm.

## 3. Experimental Results and Discussions

**Figure 4:**
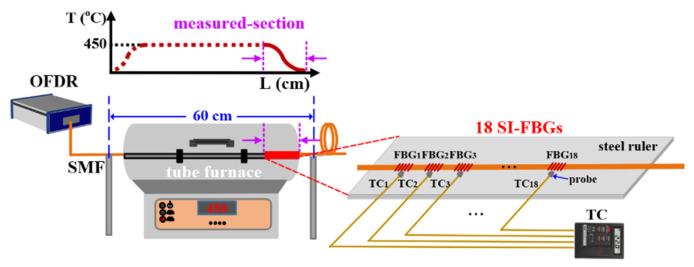
Experimental setup for annealing and quasi-distributed temperature sensing using strong SI-FBGs consisting of 18 FBGs. Inset: schematic layout diagram of the strong SI-FBGs and thermocouple (TC).

**Figure 5:**
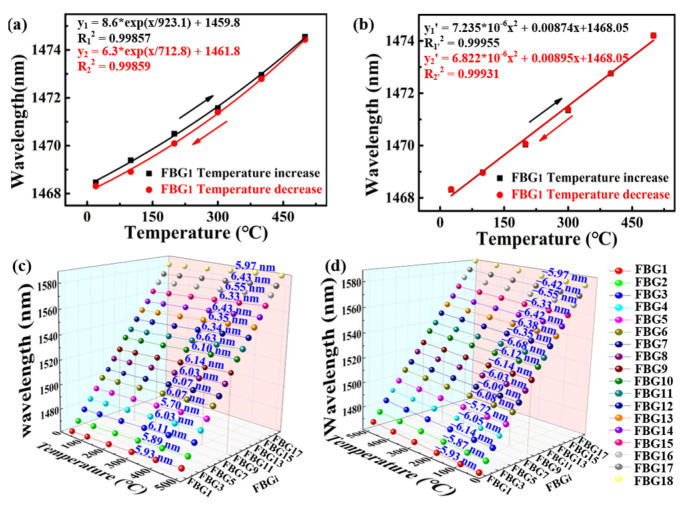
Temperature response of selected FBG_1_ of the afore-inscribed strong SI-FBGs sample (**a**) before and (**b**) after annealing for 48h with a high temperature of 500 °C; reflection wavelength shifts for strong SI-FBGs, i.e., FBG_1_ to FBG_18_, with temperature (**c**) increase and (**d**) decrease from room temperature to 500 °C in steps of 100 °C after annealing.

To investigate the temperature response of the inscribed strong SI-FBGs, a tube furnace used for annealing is shown in Figure 4, the temperature of the tube furnace was increased from room temperature to 500 °C and then decreased to room temperature in steps of 100 °C. As shown in Figure 5a, the Bragg wavelength of the selected FBG_1_ of afore-inscribed strong SI-FBGs exhibited an exponential red or blue shift with temperature increase and decrease, which was also applicable to the remained 17 FBGs. It is obvious that the change of the Bragg wavelength with the temperature increase was different from that with the temperature decrease, resulting from the residual stress in the fiber [19]. Thus, the strong SI-FBGs were annealed at the temperature of 500 °C for 48 h to release the residual stress. Compared with un-annealed strong SI-FBGs, the selected FBG_1_ exhibited almost the same wavelength shift with temperature increase and decrease, where the value of the R-square was more than 99.9%, as illustrated in Figure 5b. As shown in Figure 5c,d, the FBG_2_, FBG_3_…, and FBG_18_ exhibited the wavelength shift of 5.93, −5.93, 5.89, −5.87…, and 5.97, −5.97 nm, respectively, when the temperature was increased and decreased from room temperature to 500 °C after annealing. The almost same wavelength shifts for 18 FBGs indicated that the residual stress in the fiber was completely relaxed after the annealing process. Therefore, the fitting function of each FBG between the reflection wavelength shift and temperature could be established.

**Figure 6:**
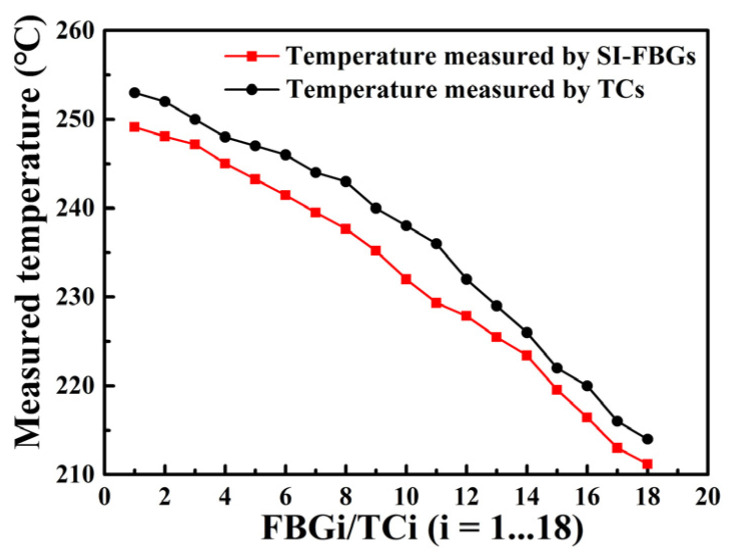
Obtained temperature gradient fields of the section for the tube furnace labeled by red in Figure 4 using strong SI-FBG and TC, respectively.

As we know, the temperature in the middle section of the tube furnace was stable, while the temperature at the edge had a certain gradient. Therefore, the afore-annealed strong SI-FBGs with a length of 17.4 mm were employed to measure the gradient temperature field of the edge of the tube furnace. As shown in the inset in Figure 4, the strong SI-FBGs and 18 thermocouples (TCs) were fixed correspondingly on the steel ruler to measure the gradient temperature field of the section labeled by red in Figure 4 when the temperature was increased to 450 °C and maintained for 2 h. Note that the 18 TCs were used to measure the temperature for reference. As shown in the inset in Figure 4, the probe position of each TC, i.e., 18 TCs, corresponds to the position of each FBG, i.e., 18 FBGs. According to the fitting function of each FBG in Figure 5, the temperature change could be obtained by the change of the wavelength shift. The obtained temperature gradient fields at the edge of the tube furnace measured by the SI-FBGs and TCs are illustrated in Figure 6. As shown in Figure 6, the temperatures measured by the strong SI-FBGs and TCs were much lower than the set temperature of the tube furnace, i.e., 450 °C. And the temperature field ranges measured by the SI-FBGs and TCs were 249.13–211.18 °C and 253–214 °C, respectively. The measured temperature divergence was due to the position mismatch of the strong SI-FBGs and TC, where the sizes of the SI-FBGs and TC were 470 μm and 2 mm, respectively. But the measured temperature fields using SI-FBGs and TCs exhibited the same trend, i.e., the further away from the middle-stable temperature field, the lower the temperature, which resulted from air change between the tube furnace and the outside environment. Therefore, the strong SI-FBGs could be used as the quasi-distributed sensor to measure the temperature. As shown in Figure 2b, the FBG length was 470 μm, and the space between the two adjacent FBGs was approximately 509 μm. So, a high spatial resolution of less than 1 mm was achieved in the distributed temperature sensing experiment. Moreover, the spatial resolution was dependent on the distance between the two adjacent FBGs of the SI-FBGs, i.e., the sum of the FBG length and the space between two adjacent FBGs, which could be improved by adjusting the inscription parameters. And compared with the SI-FBGs inscribed by UV laser, the SI-FBGs inscribed by femtosecond laser also could be used for distributed high-temperature sensing up to 1000 °C [20].

**Figure 7:**
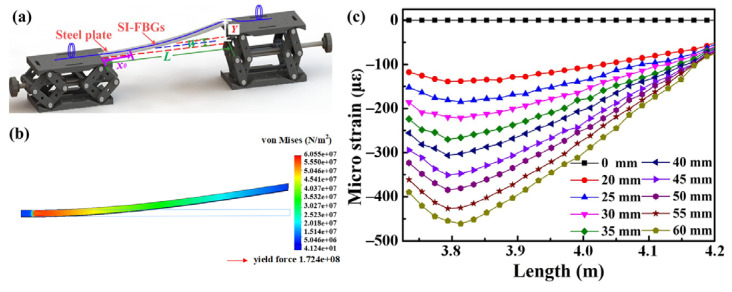
(**a**) Experimental setup, i.e., cantilever beam, to measure the distributed strain when the right end was gradually lifted to the height of *h* using the weak SI-FBGs; (**b**) simulation result of the strain distribution obtained by the SOLIDWORKS when the applied force was 1 N, i.e., *F* = 1 N to cantilever beam; (**c**) measured strain distribution for the cantilever beam using the weak SI-FBG.

To investigate the strain response of the afore-inscribed weak SI-FBGs, the experimental setup, i.e., the cantilever beam, illustrated in Figure 7a, was built up. Firstly, the weak SI-FBGs were fixed on the upper surface of the steel plate using the epoxy and maintained for 24h to make it solidify. The length, width, and height of the steel plate were *L* = 475 mm, *W* = 20 mm, and *H* = 1.5 mm, respectively. As shown in Figure 7a, the left end fixed on the lifting platform was kept at the initial height, while the right end, i.e., the free end, was gradually raised in steps of 5 mm to apply the distributed strain. When the free end of the cantilever beam was subjected to the force of *F*, the deflection, i.e., *Y*, would occur.

Thus, the strain of the cantilever beam at each point would change, and the strain at the position of *x_0_*, i.e., $\varepsilon_{x_{0}}$ could be given by [21]

(1) $$\varepsilon_{x_{0}}=\frac{3\left(L-x_{0}\right)HY}{2L^{3}},$$

where *L* and *H* are the length and height of the cantilever beam, respectively. The relationship between the subjected force, i.e., *F*, and the deflection, i.e., *Y*, could be given by

(2) $$Y=FL^{3}/3EI,$$

where *E* and *I* are the elastic modulus and moment of the inertia of the cantilever beam, and $I=BH^{3}/12$.

Substituting Equation (2) to Equation (1), the strain at the position of *x_0_* could be given by

(3) $$\varepsilon_{x_{0}}=6\left(L-x_{0}\right)F/EBH^{2}.$$

When the strain was applied to an FBG with a reflection wavelength of *λ_B_*, the wavelength shift, i.e., $\Delta \lambda_{B}$, could be given by

(4) $$\Delta \lambda_{B}=\lambda_{B}\left(1-P_{e}\right)\varepsilon_{x_{0}},$$

where *P_e_* is the coefficient of the elastic-optic, and *P_e_* = 0.22 for the SMF. Substituting Equation (3) to Equation (4), we could obtain

(5) $$\Delta \lambda_{B}/\lambda_{B}=4.68\left(L-x_{0}\right)F/\left(EBH^{2}\right).$$

Equation (5) indicated that the wavelength shift, i.e., $\Delta \lambda_{B}$, was proportional to the applied force, i.e., *F*. In addition, the closer the FBG was to the left end of the cantilever beam, the greater the wavelength shift. Therefore, the strain distribution of the cantilever beam obtained by the SOLIDWORKS 2018 USA is illustrated in Figure 7b according to the Equation (5), when the applied force of the free end was 1 N, i.e., *F* = 1 N. It is obvious that the minimum and maximum von Mises were 41.24 and 6.055e^7^ N/m^2^, and the corresponding raised heights were 0 and 28.31 mm, respectively. The applied strain of each FBG for the weak SI-FBGs could be calculated by Equation (4), i.e., the wavelength shift measured by the OFDR when the free end of the cantilever beam was raised to different heights. As shown in Figure 7c, the strain on the cantilever beam was even, i.e., 0 με, measured by the weak SI-FBGs, when the free end was at the initial height. The distribution of the applied strain on the cantilever beam exhibited the same trend, i.e., the maximum strain occurred at the position of approximately 3.8 m and gradually decreased towards both ends when the free end was raised from 20 to 60 mm in steps of 5 mm. The uneven strain distribution measured by the weak SI-FBGs agreed well with the simulation result. As shown in Figure 3b, the length of the single FBG was 1 mm with an equal space of 20 mm between the two adjacent FBGs. So, a spatial resolution of approximately 21 mm was achieved in the distributed strain sensing experiment. The spatial resolution depends strongly on the distance between the two adjacent FBGs. Thus, the spatial resolution of the strain sensing also could be improved to less than 1 mm by decreasing the distance between the two adjacent FBGs. However, the reflection bandwidth of FBG will become wider with the decrease of the FBG length, which is not conducive to the wavelength demodulation for sensing [22]. Moreover, the spatial resolution is also limited by spectral shadows and multiple reflections caused by the length of each FBG and the space between two adjacent FBGs [23]. Therefore, the spatial resolution should be adjusted according to the actual requirement.

## 4. Conclusions

An SI-FBGs inscribing system based on the femtosecond laser point-by-point technology was demonstrated to continuously inscribe a series of FBGs, i.e., SI-FBGs, with different or identical grating pitches. The strong SI-FBGs consisting of 18 FBGs with different grating pitches were successfully inscribed to measure the temperature gradient field in the tube furnace, in which a high spatial resolution of less than 1 mm was achieved. Moreover, the weak SI-FBGs consisting of 60 FBGs with identical grating pitches were successfully inscribed to measure the uneven strain distribution on the cantilever beam. Compared with the UV laser phase mask method, the femtosecond laser point-by-point technology could be used to inscribe strong and weak SI-FBGs with an adjustable pitch in almost all types of fibers with or without photosensitivity. Moreover, the fiber coating was not stripped off before the FBG inscription, which improved the robustness of the FBG sensor. Hence, our high-quality strong and weak SI-FBGs could be developed to be various quasi-distributed sensors with a high spatial resolution.

## Data Availability

Data underlying the results presented in this paper are not publicly available at this time but may be obtained from the authors upon reasonable request.
